# A Case Report of a Young Female With Renal Infarction Secondary to Breakthrough COVID Infection

**DOI:** 10.7759/cureus.25527

**Published:** 2022-05-31

**Authors:** William A Vasquez Espinosa, Andrea Santos Argueta, Vanessa A Hurtado Tandazo, Carla F Vasquez Espinosa

**Affiliations:** 1 Internal Medicine, Saint Peter’s University Hospital, New Brunswick, USA; 2 Internal Medicine, University of Miami Miller School of Medicine, Jackson Memorial Hospital, Miami, USA; 3 General Practice, Universidad Tecnológica Equinoccial, Quito, ECU

**Keywords:** : acute kidney injury, antiphospholipid antibodies, hypercoagulable state, covid19 infection, renal infarct

## Abstract

COVID-19 infection is a disease that induces a hypercoagulable state that appears to be more aggressive than other conditions related to endothelial damage. The kidney, a highly vascularized organ rich in Angiotensin-Converting Enzyme 2 (ACE2) receptors, is commonly affected by COVID-19 infection. Acute kidney injury (AKI) is common in these patients and has been linked to worse outcomes. Furthermore, kidney infarction, although uncommon, has also been reported. We present the case of a 21-year-old otherwise healthy female presenting with flank pain who was found to have renal infarction in the setting of breakthrough COVID-19 infection and Oral contraceptive pill (OCP) use. Despite getting appropriate vaccination, the patient was infected. She was not hypoxic, and her kidney function was preserved. CT angiography demonstrated peripheral hypoattenuation in the right kidney compatible with infarct but no evidence of a thrombus. The patient was medically managed with anticoagulation, and supportive therapy was offered for pain control. She had clinical improvement. The follow-up at three weeks showed normal renal function. She was continued with novel oral anticoagulation (NOAC). This case demonstrates that COVID-19 infection may present renal infarction in otherwise healthy young individuals even after appropriate vaccination. Early recognition is essential so that appropriate therapy can be given. Long-term anticoagulation and outcomes of this entity must be studied.

## Introduction

Since the pandemic, COVID-19 infection has been related to a hypercoagulable state. Deep venous thrombosis (DVT) and pulmonary embolism (PE) were identified early during the pandemic, and anticoagulation therapy was proposed for COVID-19 patients. Anticoagulation became one of the cornerstones of COVID-19 therapy. Several studies have found that this hypercoagulable state is multifactorial and linked with endothelial dysfunction, excessive cytokine release, complement activation, direct cell damage, and direct coagulation cascade activation by the virus. Patients with COVID-19 may develop large vessel thrombosis as well as thrombotic microangiopathy. Unfortunately, studies have shown that thrombosis may occur even when patients are on appropriate anticoagulation [3].

SARS-COV2 enters the human cells through the ACE2 receptors, and these receptors are expressed in large amounts in the renal tissue. Studies have shown that in special AKI pathology, kidney damage is related to a local and systemic inflammatory and immune response. The endothelial dysregulation plays a pivotal role in the pathophysiology of thromboembolism [3]. Renal infarction has also been linked with COVID infection in several studies. In most cases, it presents in a different age group, around 60 years old, and it is also more prevalent in cases of unvaccinated patients with severe COVID-19 illness. However, in our case, we saw that a young patient with no comorbidities might also develop renal infarcts [2]. 

## Case presentation

**Figure 1:**
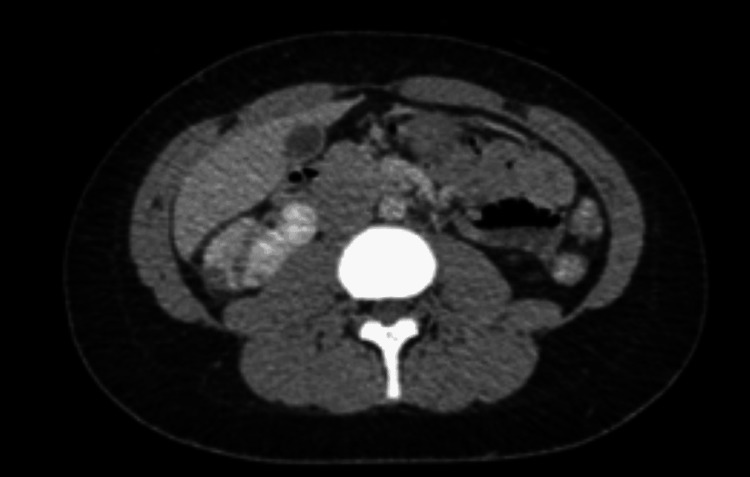
Right kidney hypoattenuations consistent with renal infarct.

A 21-year-old Hispanic female with no significant medical history presented to the emergency department with right-sided flank pain, accompanied by non-bloody diarrhea for five days. She did not report urinary symptoms, fever, or changes to her periods. The patient had no history of complicated urinary tract infections or passed stones. Of note, she reported that her coworkers were diagnosed with COVID-19 one week prior to presenting at the ER. She received three doses of the Pfizer vaccine. She was not hypoxic. Her vitals were temperature of 100.3F, BP 125/79, HR 93, and RR of 14, saturating 99% on RA. SARS-COV2 RNA PCR was positive. The rest of the basic workup was unremarkable, except for mild creatinine elevation (0.84, baseline 0.67) (table 1). Her urinalysis showed low spec gravity of 1.004 but otherwise was normal (table 2). A CT scan of the abdomen and pelvis was obtained. It showed multiple sub-centimeter areas of hypodensity along the periphery of the mid to lower pole of the right kidney compatible with renal infarction (figure 1).

**Table 1:** CBC, BMP at admission, discharge and two months after discharge. CBC- Complete blood count; BMP- basic metabolic panel; WBC- White blood cells; RBC: Red blood cells; Hb- Hemoglobin; Hct- hematocrit; MCV- mean corpuscular volume; BUN- blood urea nitrogen; eGFR- estimated glomerular filtration rate

		Admission	Discharge	Two Months After Discharge
Complete blood count	WBC	9.4 x 10^9^/L	6.3 x 10^9^/L	6.4 x 10^9^/L
	RBC	4.73 x 10^12^/L	4.41 x 10^12^/L	4.65 x 10^12^/L
	Hb	14.5 g/dL	13.3 g/Dl	13.8 g/dL
	Hct	41.5%	38.9%	40.1%
	MCV	87.8 fL	88.2 fL	87.6 fL
	Platelet	381 x 10^9^/L	347 x 10^9^/L	282 x 10^9^/L
	Neutrophiles	64%	45.2%	59.5%
	Lymphocytes	16.1%	38.9%	29.8%
	Monocytes	17.8%	12.6%	7.8%
	Eosinophils	1.2%	2.4%	1.8%
	Basophils	0.9%	0.9%	1.1%
Blood chemistry	BUN	9 mg/dL	16 mg/dL	13 mg/dL
	Creatinine	0.84 mg/dL	0.67 mg/dL	0.67 mg/dL
	eGFR	85 mL/min/1.73	111 mL/min/1.7	111 mL/min/1.7
	Sodium	135 mmol/L	136 mmol/L	136 mmol/L
	Potassium	3.7 mmol/L	4.4 mmol/L	4.2 mmol/L
	Chloride	99 mmol/L	104 mmol/L	105 mmol/L
	Bicarbonate	25 mmol/L	23 mmol/L	23 mmol/L
	Anion Gap	11	9	8

**Table 2:** Urinalysis on admission.

Result name	Result
Color	Yellow
Appearance	Clear
Urine glucose	Negative
Urine bilirubin	Negative
Ketones	Negative
SP Gravity	1.004
Urine Blood	Moderate
Urine pH	6
Protein	Negative
Urobilinogen	<2 mg/dL
Nitrites	Negative
Leukocyte esterase	Negative
Ascorbic acid	Negative
WBC	0-2 cells/hpf
Red blood cells	0-2 cells/hpf
Epithelial	0-2 cells/hpf

The patient was asked about further risk factors for renal infarction. She did not have a family history of coagulation disorders, never smoked, did not have any trauma to the affected area, had no surgeries, and had no history of malignancy. However, the patient was taking OCPs, they were held, and she was started on anticoagulation with heparin. Further studies were sent, which included fibrinogen, prothrombin gene mutation, factor V Leiden, factors S&C, Anti-Thrombin III, homocysteine, lupus anticoagulant, and antinuclear antibody (ANA). All of them were normal. Patient D-dimer was less than 150ng/ml, and venous Doppler of lower extremities was negative for DVT. An echocardiogram was also obtained, and it did not show vegetations but demonstrated patent foramen ovale (PFO). Cardiac monitoring and EKG did now show arrhythmias.

A CT angiography was obtained to evaluate the infarct and possible revascularization further. It showed peripheral hypoattenuations in the right kidney compatible with infarction. However, no evidence of a thrombus as such patient was managed medically with anticoagulation that was changed to therapeutic doses of enoxaparin (60mg twice daily) while in the hospital and then discharged on apixaban 10 mg twice daily for one week; later reduced to 5mg twice daily. Her hospital course was uncomplicated.

Holter's monitoring as an outpatient did not show any arrhythmias, and her follow-up BMP and UA at three weeks and two months were unremarkable, and GFR was preserved. She is still on anticoagulation and following up with her hematologist.

## Discussion

Renal infarction is a rare condition usually in individuals suffering from hypercoagulable states or with a history of atrial fibrillation or conditions predisposing to embolic events. The clinical presentation of a renal infarct usually includes flank or abdominal pain, and the pain is usually localized and may be accompanied by nausea, vomiting, chills, fever, and hematuria. These symptoms may confuse the clinicians, and the renal infarct is often misdiagnosed as a renal stone, pyelonephritis. Luckily, renal infarcts are easily detectable after obtaining CT scans with imaging technologies. Renal infarcts are usually unilateral. However, bilateral cases have been reported and are linked to hypercoagulable states [1]. 

Once a renal infarct is detected. High suspicion should be put on a cardioembolic event, as this is the primary mechanism of infarction. An EKG should be obtained as soon as possible to try to detect atrial fibrillation. As it is well known, there is a high likelihood that arrhythmia will not be seen on a plain EKG as such continuous heart monitoring is required. The risk factors for atrial fibrillation should be assessed for every patient. Our patient had a low risk of having atrial fibrillation, which prompted us to consider a hypercoagulable state as the mechanism of pathology [2].

SARS-COV-2 causes endothelial dysregulation, which plays a pivotal role in the pathophysiology of thromboembolism in COVID-19 infection [3]. As a result, thrombosis may happen in the arterial and venous circulation. Evidence suggests renal infarction is a possibility in COVID-19 infected patients. However, it has been exclusively seen in an unvaccinated population and severe diseases [4].

Our young female was indeed predisposed to having clots while using OCPs, and her COVID infection likely constituted a second hit to her already hypercoagulable state. Regardless, a further workup for hypercoagulable states should be pursued in patients like this. Genetic disorders and autoimmune conditions were evaluated. In our patient, we did not find any abnormal results when checking for fibrinogen, prothrombin gene mutation, factor V Leiden, factors S&C, anti-thrombin III, homocysteine, lupus anticoagulant, antiphospholipid antibodies, or ANA. Of note, when COVID-19 infection has been involved in renal infarcts, antiphospholipid antibodies may be positive. However, the role of these antibodies is still unclear, and they are not always present [5]. 

After the infarct is detected, it is vital to evaluate the extent of the disease. The renal infarcts are usually localized to a kidney region and are unilateral, thus having a good prognosis. The unaffected kidney can overcome the insult and maintain renal function. However, several degrees of renal failure may be seen in some cases, and immediate assessment and treatment are crucial in preventing irreversible damage [6].

We usually obtain a computed tomography angiography (CTA) for this evaluation to see if the patient may benefit from revascularization depending on the affected renal territory. If a thrombus is seen, the patient may be amenable to endovascular therapy. This procedure should be done in patients in which renal recovery is expected. The best outcomes in patients with renal infarcts are seen in acute infarcts (onset of symptoms less than six hours), main renal artery involvement, or vascular dissection. Patients in which endovascular therapy is not pursued should be anticoagulated [7]. 

In our patient, we saw peripheral hypoattenuation on her right kidney involving a wedge-shaped area pointing toward a subacute infarct involving a subsegmental artery. Her kidney function was preserved as such medical therapy was pursued. She was started on anticoagulation. It is unclear for how long anticoagulation should be maintained in these kinds of patients. Patients with renal infarct usually are anticoagulated for at least three months to a lifetime. In our patient, her risk factors predisposing thrombosis were COVID and OCPs. The OCPs were discontinued right away, and alternative nonhormonal anticonception was advised.

Furthermore, we expect that her hypercoagulable state would reverse outside the period of acute illness from COVID. We advised at least three months of anticoagulation to our patient and close follow-up with her hematologist, regardless of the length of anticoagulation. This therapy has been related to improved survival and better outcomes [8].

Follow-up of patients with renal infarction is needed as long-term complications such as hypertension, or worsening eGFR may ensue. On admission, our patient had decreased GFR, which returned to her normal baseline on discharge and was preserved at three months. 

## Conclusions

Thromboembolic complications of COVID-19 are well documented. Various mechanisms have been proposed to explain this finding. The kidney is commonly affected by COVID-19 infection, and tissue inflammation and local immune cell infiltration might have a critical role in kidney injury, as might endothelial damage and microvascular thrombi. Isolated renal infarction as the initial presentation of COVID-19 has been reported. Patients with renal infarcts benefit from prompt recognition of this pathology and appropriate treatment that may involve endovascular therapy or anticoagulation. The etiology of the infarct should be investigated, and Cardioembolic phenomena and hypercoagulable states should be considered. After a complete evaluation, anticoagulation therapy should be given. The length of anticoagulation is unclear for patients with COVID and renal infarct and should be decided on a case-by-case basis.
